# Inducible degradation of dosage compensation protein DPY-27 facilitates isolation of *Caenorhabditis elegans* males for molecular and biochemical analyses

**DOI:** 10.1093/g3journal/jkac085

**Published:** 2022-04-11

**Authors:** Qianyan Li, Arshdeep Kaur, Benjamin Mallory, Sara Hariri, JoAnne Engebrecht

**Affiliations:** 1 Department of Molecular and Cellular Biology, University of California, Davis, Davis, CA 95616, USA; 2 Biochemistry, Molecular, Cellular and Developmental Biology Graduate Group, University of California, Davis, Davis, CA 95616, USA

**Keywords:** *Caenorhabditis elegans*, dosage compensation, DPY-27, males, meiosis, spermiogenesis, Genetics of Sex

## Abstract

Biological sex affects numerous aspects of biology, yet how sex influences different biological processes have not been extensively studied at the molecular level*. Caenorhabditis elegans*, with both hermaphrodites (functionally females as adults) and males, is an excellent system to uncover how sex influences physiology. Here, we describe a method to isolate large quantities of *C. elegans* males by conditionally degrading DPY-27, a component of the dosage compensation complex essential for hermaphrodite, but not male, development. We show that germ cells from males isolated following DPY-27 degradation undergo meiosis and spermiogenesis like wild type and these males are competent to mate and sire viable offspring. We further demonstrate the efficacy of this system by analyzing gene expression and performing affinity pull-downs from male worm extracts.

## Introduction

In metazoans, sex has evolved multiple times and influences most biological processes. Gonochoristic species have 2 biological sexes, defined by the production of differentiated gametes: sperm (males) and ova (females). Somatic tissues also display sexually dimorphic features, the most obvious being those important for mating. Furthermore, studies in mammals have highlighted the impact of sex on physiological processes including metabolism, cardiac, and neuronal functions (Miller 2014). The molecular mechanisms underlying how biological processes are modulated by sex remain largely unknown.

In addition to gonochorism, other reproductive strategies exist. For example, hermaphrodism is common in many species including snails, worms, echinoderms, fish, and plants, where both sperm and ova are produced in the same organism. The nematode *Caenorhabditis elegans* is an androdioecious species with both hermaphrodites and males and has been an excellent model to study sex-specific morphological adaptations and production of sperm and ova. In *C. elegans* hermaphrodites (*XX*), the first wave of germ cells undergoes spermatogenesis; as adults, hermaphrodites exclusively produce ova and thus are functionally female (Hubbard and Greenstein 2000). Males (*XO*) arise spontaneously at a low frequency (∼0.2%) because of meiotic chromosome nondisjunction and exclusively produce sperm. The morphological features that differentiate the *C. elegans* male from the hermaphrodite arise during postembryonic development. Most prominent is the tail structure and associated male-specific neuronal wiring required for mating (Sulston *et al.* 1980). Males can be propagated by crossing with hermaphrodites, which will preferentially use male sperm to fertilize ova, leading to a 1:1 hermaphrodite: male ratio in the offspring (Ward and Carrel 1979; LaMunyon and Ward 1995; LaMunyon and Ward 1998).

*Caenorhabditis* *elegans* is a facile genetic model due in part to its facultative hermaphroditic lifestyle, which greatly simplifies genetic analysis. The wealth of genetic mutants available and in-depth understanding of how sex is determined, facilitates mechanistic studies of how sex affects biological processes. For example, *fog-2* loss-of-function mutants block spermatogenesis specifically in hermaphrodites, leading to true female worms (Schedl and Kimble 1988). These mutant worms have been used to distinguish sperm vs ova contributions to euploid progeny (Jaramillo-Lambert *et al.* 2010; Checchi *et al.* 2014; Li *et al.* 2020). Several genes required for spermatogenesis (*spe* genes) have been identified. Most *spe* genes are required for spermatogenesis in both hermaphrodites and males (Nishimura and L'Hernault 2010). Conditional depletion of one of these, *spe-44*, has been developed for mating and longevity studies (Kasimatis *et al.* 2018). Interestingly, the *spe-8* group is specifically required for activation of hermaphrodite, but not male, sperm (L'Hernault *et al.* 1988). Additionally, mutations in genes that are important for *X* chromosome disjunction in meiosis leads to the Him (High incidence of males) phenotype, such that up to 40% of self-progeny are males (Hodgkin *et al.* 1979).

*Caenorhabditis* *elegans* has prominent gonads where germ cells are organized in a linear assembly line fashion and reproduces prolifically, making *C. elegans* an outstanding system to investigate meiosis and fertilization. To date, most meiotic studies have focused on oogenesis in hermaphrodites due to the ease of isolating meiotic mutants and performing molecular analyses. A few studies focusing on male meiosis have revealed that while the basic processes are similar, the regulation of meiosis is distinct in spermatogenesis vs oogenesis (Jaramillo-Lambert *et al.* 2007; Shakes *et al.* 2009; Jaramillo-Lambert *et al.* 2010; Checchi *et al.* 2014; Kurhanewicz *et al.* 2020). However, a complete understanding of how sex influences meiosis, gametogenesis, and biology more generally is still lacking.

While hermaphrodites are easy to propagate and collect in large numbers, it has been more difficult to propagate and collect large numbers of males. Most strategies rely on maintaining mated cultures (∼50% males) or using *him-8* mutants (∼40% males; Hodgkin *et al.* 1979) and then manually picking males. Alternatively, males can be separated from hermaphrodites using filters, which take advantage of the different size of adult hermaphrodites (1 mm × 80 µm) and males (0.8 mm × 50 µm; Chu *et al.* 2006). However, this requires synchronized cultures as larvae are smaller and will filter with males regardless of sex. Additionally, filtering requires extensive labor and in our hands is not very efficient, making it difficult to collect large quantities required for biochemical analyses.

Here, we describe a new method to isolate relatively pure populations of males in large numbers. This method takes advantage of the inducible degradation of DPY-27, a component of the dosage compensation complex (DCC; Plenefisch *et al.* 1989). In *C. elegans*, the DCC downregulates gene expression from the 2 *X* chromosomes in hermaphrodites such that the overall level is comparable to the expression from the single *X* chromosome in males. Consequently, the DCC is essential for embryonic development in hermaphrodites but not in males. Worms defective for the DCC are therefore hermaphrodite-specific lethal (Meyer 2005). Using the auxin-inducible degradation system that has been adapted for *C. elegans* (Zhang *et al.* 2015; Ashley *et al.* 2021), we fused a degron tag to DPY-27 and constructed strains also expressing TIR1 in the *him-8* mutant background, which produces male self-progeny (Hodgkin *et al.* 1979). We show that males collected after auxin treatment exhibit normal meiosis and spermiogenesis and that these males are proficient for mating and sire viable progeny. We demonstrate the effectiveness of this method by analyzing gene expression and performing affinity pull-downs followed by mass spectrometry from male worm extracts.

## Materials and methods

### Genetics

*Caenorhabditis* *elegans* var. Bristol (N2) was used as the wild-type strain. The following strains were constructed:

JEL1197: *sun-1p::TIR1 II; dpy-27::AID::MYC (xoe41) III; him-8(me4) IV*

JEL991: *sun-1p::TIR1 II; dpy-27::AID::MYC(xoe41) brd-1::gfp::3xflag(xoe14) III; him-8(me4) IV*

JEL1217: *sun-1p::TIR1 II; gfp(glo)::3xflag::cosa-1(xoe44) dpy-27::AID::MYC (xoe41) III; him-8(me4) IV*

JEL1214: *mex-5p::TIR1 I; dpy-27::AID::MYC (xoe41) III; him-8(me4) IV*

*sun-1p::TIR1* and *mex-5p::TIR1* strains were graciously provided by Jordan Ward (UCSC; Ashley *et al..* 2021). Some nematode strains were provided by the Caenorhabditis Genetics Center, which is funded by the National Institutes of Health National Center for Research Resources (NIH NCRR). Strains were maintained at 20°C.

### CRISPR-mediated generation of alleles

*dpy-27::AID::MYC(xoe41)* and *gfp(glo)::3xflag::cosa-1(xoe44)* were generated using the co-CRISPR method as described (Paix *et al.* 2015). GermLine Optimized GFP sequence was used to enhance germline expression and prevent potential silencing (Fielmich *et al.* 2018). Guide sequence, repair template, and genotyping primers are provided in Supplementary File 1. Correct editing was verified by Sanger sequencing. Worms generated by CRISPR were outcrossed a minimum of 4 times.

### Auxin treatment to generate male cultures

Synchronized L1 larvae were placed on NGM plates containing 1 mM auxin (Naphthaleneacetic Acid; K-NAA; PhytoTech #N610) and maintained at 20°C until adulthood. Worms were washed off plates and bleached in a final concentration of 0.5 N NaOH and 1% bleach (Porta-de-la-Riva *et al.* 2012). Embryos were collected by centrifugation at 1,300 g for 1** **min and washed 3× with M9 buffer supplemented with 1 mM auxin. Embryos were transferred to a new tube with M9 buffer + 1 mM auxin and kept on a rocker for 24 h. L1 larvae together with unhatched embryos were washed with M9 buffer and plated onto NGM plates without auxin. Approximately two and half days later, viable worms were quantified for % males and used for downstream analyses. A detailed protocol for large-scale collection of male worms is provided in Supplementary File 2.

### Cytological analyses

Immunostaining of germlines was performed as described (Jaramillo-Lambert *et al.* 2007) except slides were fixed in 100% ethanol instead of 100% methanol for direct GFP fluorescence of GFP*::*COSA-1. Rabbit anti-RAD-51 (1:10,000; cat #2948.00.02; SDIX; RRID: AB_2616441), mouse anti-Tubulin (1:500; cat# T9026, Sigma-Aldrich; RRID: AB_477593), and secondary antibodies Alexa Fluor 488 donkey anti-mouse and 594 donkey anti-rabbit IgG (1:500) from Life Technologies were used. DAPI (2 µg/ml; Sigma-Aldrich) was used to counterstain DNA.

Collection of fixed images was performed using an API Delta Vision Ultra deconvolution microscope equipped with a 60×, NA 1.49 objective lens, and appropriate filters for epi-fluorescence. Z-stacks (0.2 µm) were collected from the entire gonad. A minimum of 3 germlines was examined for each condition. Images were deconvolved using Applied Precision SoftWoRx batch deconvolution software and subsequently processed and analyzed using Fiji (ImageJ) (Wayne Rasband, NIH).

RAD-51 foci were quantified in germlines of age-matched males (18–24 h post-L4). Germlines were separated into the transition zone (leptotene/zygotene), as counted from the first and last row with 2 or more crescent-shaped nuclei, and pachytene, which was further divided into 3 equal parts: early, mid, and late pachytene. RAD-51 foci were quantified from half projections of the germlines. The number of foci per nucleus was scored for each region.

GFP*::*COSA-1 foci were quantified from deconvolved 3D data stacks; mid-late pachytene nuclei were scored individually through z-stacks to ensure that all foci within each individual nucleus were counted.

The number of spermatocytes at metaphase and anaphase, which were identified by spindle shape and orientation (anti-Tubulin) in combination with chromosome morphology (DAPI), in the division zone were quantified.

Spermiogenesis was monitored by releasing sperm into sperm medium (50 mM HEPES, 25 mM KCl, 45 mM NaCl, 1 mM MgSO_4_, 5 mM CaCl_2_, 10 mM Dextrose; pH 7.8) in the absence and presence of 200 µg/ml Pronase E (MedChemExpress HY-114158A), 0.4 µg/ml Trypsin (Promega V511A), 1 mM ZnCl_2_ (Singaravelu *et al.* 2011; Liu *et al.* 2014), and imaged on a Leica DM4B microscope with differential interference contrast optics and a 63× Plan Apo 1.40 NA objective, a Leica K5 cMOS camera, and Leica Application Suite X version 3.7 software. Percent sperm activation (activated sperm/total) was counted from sperm released from a minimum of 10 worms.

### Analyses of male-sired progeny and sperm competitiveness

A single *fog-2(q71)* female and a single male of indicated genotype/condition were allowed to mate for 16 h on small *Escherichiacoli* OP50 spots and then the female was transferred to new plates every 24 h up to 96 h. The total number of progeny (eggs + worms) from 6 mated females was scored 24 h after removing the female. Viability of male-sired progeny was determined by mating a single *fog-2(q71)* female with 3 males of indicated genotypes/conditions on small *E. coli* OP50 spots for 24 h. The mated female was transferred to new plates every 24 h. Progeny viability was determined over 3 days by counting eggs and hatched larvae 24 h after removing the female and calculating % as larvae/(eggs + larvae). The progeny of a minimum of 8 mated females were scored.

To test sperm competitiveness, late L4 *unc-119(ed3)* hermaphrodites and males of indicated genotypes/conditions were allowed to mate for 16 h. Hermaphrodites were transferred to fresh plates every 24 h, and upon reaching adulthood, offspring were scored as either Unc (self) or non-Unc (cross) progeny and counted (Hansen *et al.* 2015).

### RT-PCR

Total RNA was isolated from 50 to 100 µl of packed worms from indicated genotypes/conditions using the RNeasy Mini Kit (Qiagen, Catalog #74104) and QIAshredder (Qiagen, Catalog #79654). One microgram of RNA was converted to cDNA using SuperScript III First-Strand Synthesis System for RT-PCR (Invitrogen, Catalog #18080-051) primed with Oligo(dT)_20_. qPCR reactions were prepared with SsoAdvanced Universal SYBR Green Supermix (Bio-Rad, Catalog #1725271) using cDNA and the following primers (final concentration, 400 nM): *ama-1* (Housekeeping) Fwd: 5′GACGAGTCCAACGTACTCTCCAAC-3′, Rev: 5′TACTTGGGGCTCGATGGGC-3′; *vit-2* (female-enriched) Fwd: 5′GCCAGAAGAACCAGAGAAGCC-3′, Rev: 5′--TGTTGTTGCTGCTCGACCTC-3′; *sncp-1.3* (male-enriched) Fwd: 5′-TCCTTCATGCGAATGACCCG-3′, Rev: 5′-GCGCTTTGAATCTACCCAGC-3′; Cq values were determined for each primer pair and normalized to the *ama-1* control. The fold change between—or + auxin and wild type was analyzed using the 2^-ΔΔCt^ method. Raw Cq values and calculations are provided in Supplementary File 3.

### Pull-down assays

Male worms were collected following auxin treatment as described above and resuspended in H100 lysis buffer (50 mM HEPES, pH 7.4, 1 mM EGTA, 1 mM MgCl_2_, 100 mM KCl, 10% glycerol, 0.05% NP-40) + protease inhibitors (Complete Ultra Tablets, Mini Protease Inhibitor Cocktail; Roche #05892791001 and 1 mM PMSF; Sigma). Worms were allowed to settle at the bottom of the tube and the lysis buffer was removed until there was 0.5–1 ml buffer covering the worm pellet. Worms were resuspended and flash frozen as “worm popcorns” by pipetting into liquid nitrogen. Worm popcorns were ground into fine powder using a SPEX SamplePrep 6970 FreezerMill and immediately stored at -80°C.

Approximately 1 ml of worm powder was thawed on ice and brought to 4 ml with H100 + protease inhibitors. The lysate was passed through a glass tissue grinder multiple times on ice and then centrifuged at 13,000 rpm for 20 min at 4°C to remove insoluble debris. Fifty microliters of anti-FLAG M2 magnetic beads (Millipore Sigma M8823) were prepared for each pull-down assay by washing with 1 ml H100 + protease inhibitors 4× using a magnetic rack separator. The soluble fraction was incubated with the washed beads for 3 h with constant rotation at 4°C. Beads were washed with 1 ml lysis buffer + protease inhibitors 4× and then washed in lysis buffer without protease inhibitors 7×. Approximately 10% of beads was analyzed by immunoblot to check efficiency of pull-down and the remaining processed for mass spectrometry.

### Mass spectrometry analyses

Processing and proteomic profiling were performed at the University of California, Davis Proteomics Core Facility (https://proteomics.ucdavis.edu). Protein samples on magnetic beads were washed 4× with 200 µl 50 mM Triethyl ammonium bicarbonate (TEAB) for 20 min each at 4°C with shaking. Two micrograms of trypsin was added to the bead/TEAB mixture and the samples were digested overnight at 4°C with shaking at 800 rpm. The supernatant was then removed, and the beads washed once with enough 50 mM ammonium bicarbonate to cover. After 20 min with gentle shaking, the wash was removed and combined with the initial supernatant. The peptide extracts were reduced in volume by vacuum centrifugation and a small portion of the extract was used for fluorometric peptide quantification (Thermo Scientific Pierce). One microgram was loaded for each LC-MS (liquid chromatography–mass spectrometry) analysis.

LC-MS/MS was performed on an ultra-high-pressure nano-flow Nanoelute (Bruker Daltonics) at 40°C with a constant flow of 400 nl/min on a PepSep 150 µm × 25 cm C18 column (PepSep, Denmark) with 1.5 μm particle size (100 Å pores) and a ZDV captive spray emitter (Bruker Daltonics). Mobile phases A and B were water with 0.1% formic acid (v/v) and 80/20/0.1% ACN/water/formic acid (v/v/vol), respectively. Peptides were separated using a 30 min gradient. Eluting peptides were then further separated using TIMS (trapped ion mobility spectrometry) on a Bruker timsTOF Pro 2 mass spectrometer. Mass spectrometry data were acquired using the dda PASEF method (Meier et al., 2018). The acquisition scheme used was 100 ms accumulation, 100 ms PASEF ramp (at 100% duty cycle) with up to 10 PASEF-MS/MS scans per topN acquisition cycle. The capillary voltage was set at 1,700 V, Capillary gas temp 200°C. The target value was set at 20,000 a.u. with the intensity threshold set at 500 a.u. The m/z range surveyed was between 100 and 1,700. Precursor ions for PASEF-MS/MS were selected in real time from a TIMS-MS survey scan using a nonlinear PASEF scheduling algorithm. The polygon filter (200–1,700 m/z) was designed to cover ions within a specific m/z and ion mobility plane to select multiply charged peptide features rather than singly charged background ions. The quadrupole isolation width was set to 2 Th for m/z < 700 and 3 Th for m/z 800.

Mass spectrometry raw files were searched using Fragpipe 16.0 (Kong *et al.* 2017) against the UniProt *C.elegans*; UP000001940 database. Decoy sequences were generated, appended, and laboratory contaminates added within Fragpipe. Default search settings were used. Carbamidomethylation of cysteine residues was set as a fixed modification, and methionine oxidation and acetylation of protein N termini as variable modifications. Decoy false discovery rates were controlled at 1% maximum using both the Peptide and Protein prophet algorithms. Label-free protein quantification was performed with the IonQuant algorithm (default settings; Yu *et al.* 2020).

Search results were loaded into Scaffold (version Scaffold_5.0.1, Proteome Software Inc., Portland, OR, USA) for visualization purposes. Proteins that contained similar peptides and could not be differentiated based on MS/MS analysis alone were grouped to satisfy the principles of parsimony. Proteins sharing significant peptide evidence were grouped into clusters. The complete list of proteins identified from the control and the unique proteins identified from the BRD-1*::*GFP*::*3xFLAG pull-downs are provided in Supplementary File 4.

### Statistical analyses

**Fig. 1.**
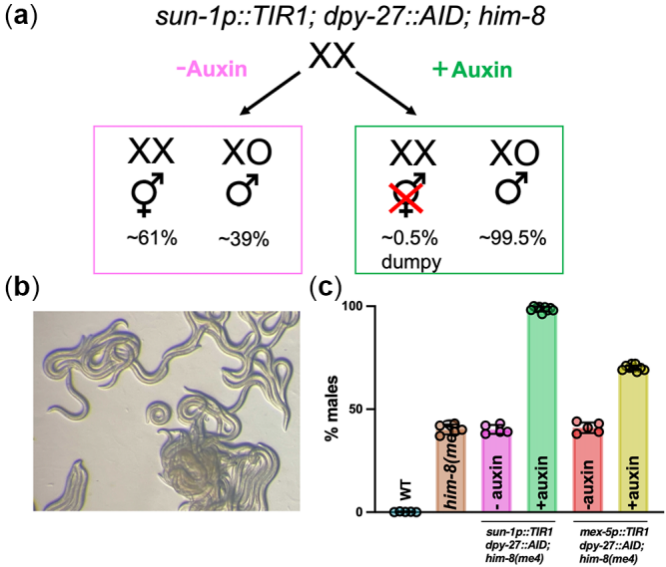
Enrichment of male worms by conditional degradation of DPY-27. a) Strategy for isolation of male cultures. b) Plate phenotype following auxin degradation of DPY-27, where greater than 98% of the worms are males. c) Quantification of male enrichment in WT, *him-8(me4)*, *sun-1p::TIR1; dpy-27::AID; him-8(me4)* and *mex-5p::TIR1; dpy-27::AID; him-8(me4)* in the absence (−auxin) and presence (+auxin) of 1 mM auxin. A minimum of 5 plates were counted for males; mean and 95% confidence intervals are shown. Statistical comparisons between + and − auxin for *sun-1p::TIR1; dpy-27::AID; him-8(me4)*: *P* = 0.001 and *mex-5p::TIR1; dpy-27::AID; him-8(me4)*: *P* = 0.0005 by Mann–Whitney.

**Fig. 2.**
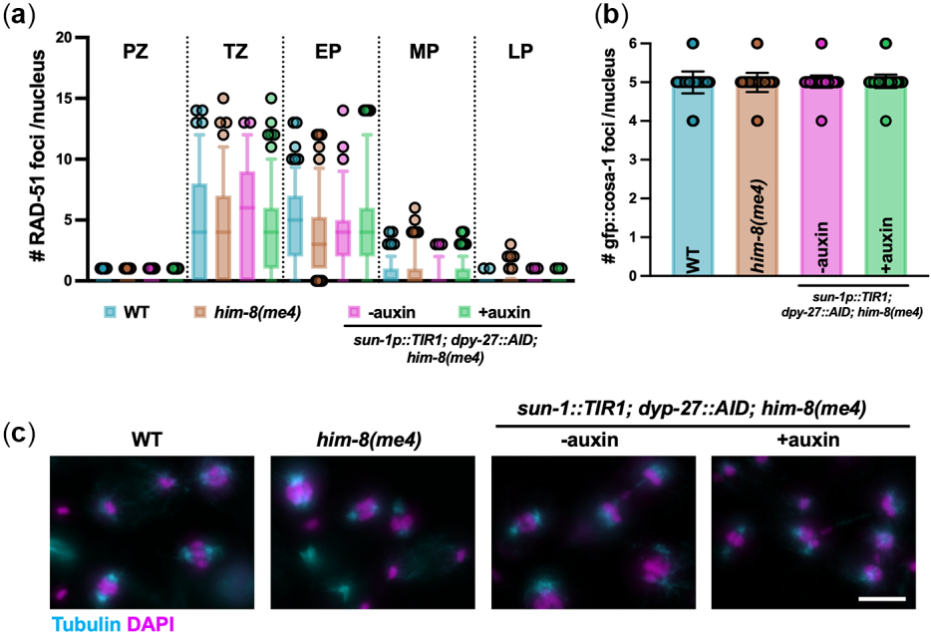
DPY-27 depletion does not affect male meiosis. a) Quantification of RAD-51 in indicated regions of the germline. Box whisker plots show number of RAD-51 foci per nucleus. Horizontal line of each box represents the median, top and bottom of each box represent medians of upper and lower quartiles, lines extending above and below boxes indicate SD, and individual data points are outliers from 5% to 95%. Comparisons by Mann–Whitney revealed no statistical differences between the strains. PZ, proliferative zone; TZ, transition zone; EP, early pachytene; MP, mid-pachytene; LP, late pachytene. Three germlines were scored for each strain/condition. Number of nuclei scored in each region: WT, PZ = 559; TZ =136; EP = 132; MP = 175; LP = 132; *him-8(me4)*, PZ = 452; TZ = 115; EP = 134; MP = 141; LP = 135; *sun-1p::TIR1; dpy-27::AID; him-8(me4)* − auxin, PZ = 391; TZ = 126; EP = 117; MP = 155; LP = 168; *sun-1p::TIR1; dpy-27::AID,; him-8(me4)* + auxin, PZ = 671; TZ = 187; EP = 197; MP = 209; LP = 206. b) Number of COSA-1 foci in mid-late-pachytene in indicated strains/conditions; mean and 95% confidence intervals are shown. Comparisons by Mann–Whitney revealed no statistical differences between the strains. Number of nuclei analyzed: wild type = 189 (from 3 worms); *him-8(me4)* = 182 (from 3 worms); *sun-1p::TIR1; dpy-27::AID; him-8(me4)* −auxin = 549 (from 8 worms); *sun-1p::TIR1; dpy-27::AID; him-8(me4)* +auxin = 584 (from 8 worms). c) Images of the division zone of male gonads labeled with anti-tubulin (cyan) to visualize the spindle and DAPI (magenta) to visualize the DNA from WT (N2), *him-8(me4)* and *sun-1p::TIR1; dpy-27::AID; him-8(me4)* – and + auxin. Scale bar = 5 microns. There was no significant difference in the number of metaphase and anaphase meiosis I and II spindles/gonad in the different genotypes/conditions (N2 = 13.5 ± 3.7; *him-8(me4)*=12.83 ± 2.1; *sun-1p::TIR1; dpy-27::AID; him-8(me4)* – auxin = 11.83 ± 2.3 and + auxin = 13.5 ± 1.9/gonad) by Mann–Whitney.

**Fig. 3.**
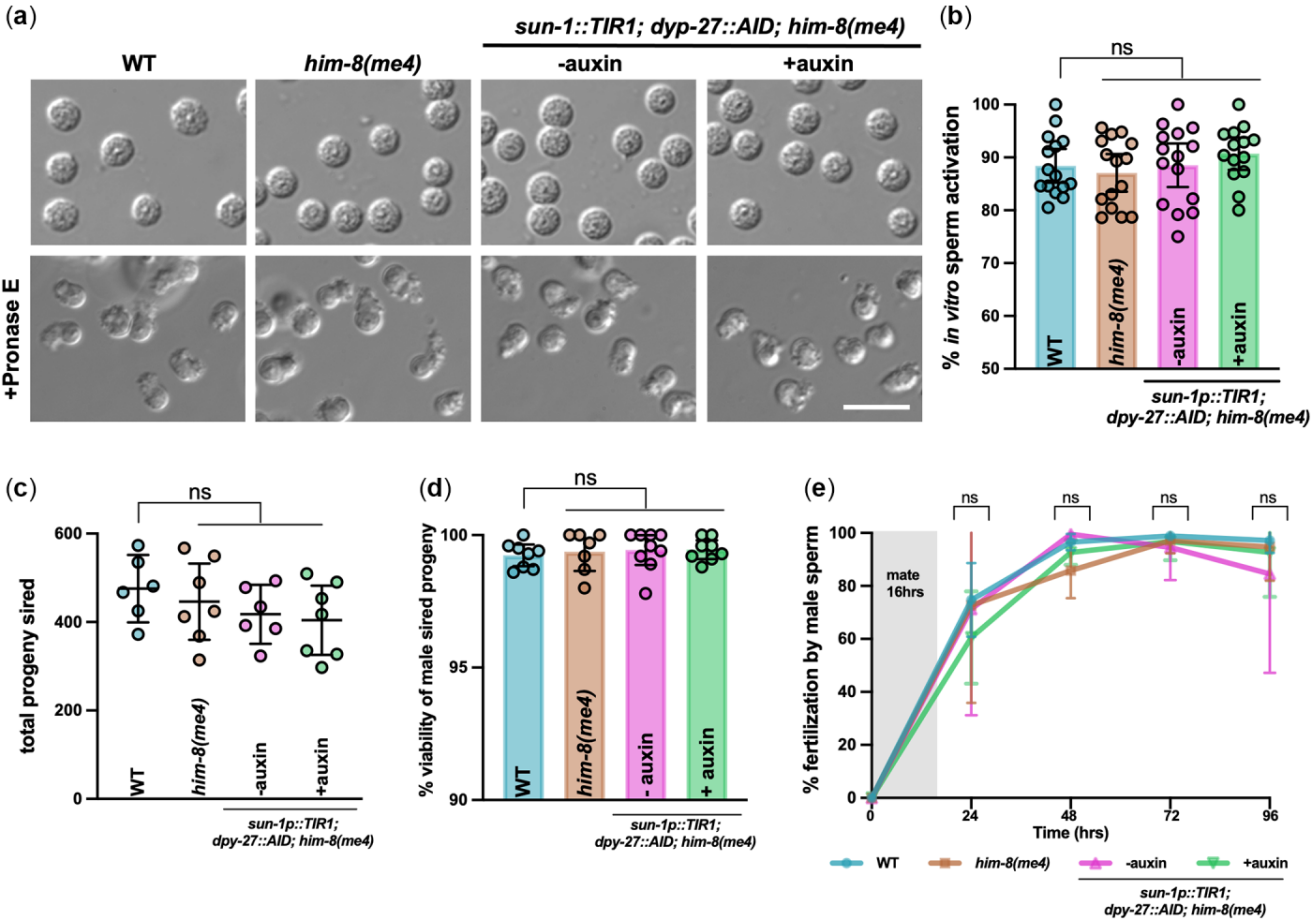
Spermiogenesis, sperm quality, and competition are not perturbed following DPY-27 degradation in males. a) Micrographs of sperm isolated from N2 (WT), *him-8(me4)*, and *sun-1p::TIR1; dpy-27::AID; him-8(me4)* −auxin and +auxin and in the presence of Pronase E (+Pronase E), which leads to sperm activation. Scale bar = 10 microns. b) Quantification of sperm activation. Comparisons by Mann–Whitney revealed no statistical differences between the strains/conditions. The number of sperm examined: N2 (WT) = 544; him-8(me4) = 1,104; *sun-1p::TIR1; dpy-27::AID; him-8(me4)* −auxin = 602; *sun-1p::TIR1; dpy-27::AID; him-8(me4)* +auxin = 647. c) Total progeny sired. Mean and 95% confidence intervals are shown. Comparisons by Mann–Whitney revealed no statistical differences between the strains/conditions. Number of crosses examined: N2 (WT) = 6; *him-8(me4)* = 7; *sun-1p::TIR1; dpy-27::AID; him-8(me4)* −auxin = 6; *sun-1p::TIR1; dpy-27::AID; him-8(me4)* +auxin = 7. d) Embryonic viability of *fog-2(q71)* progeny sired by indicated males. Mean and 95% confidence intervals are shown. Comparisons by Mann–Whitney revealed no statistical differences between the strains/conditions. Number of crosses examined: wild type = 10; *him-8(me4)* = 8; *sun-1p::TIR1; dpy-27::AID; him-8(me4)* −auxin = 10; *sun-1p::TIR1; dpy-27::AID; him-8(me4)* +auxin = 9. e) Sperm competition assays were performed with *unc-119(ed3)* hermaphrodites. Mean and 95% confidence intervals are shown. Comparisons by Mann–Whitney revealed no statistical differences between the strains/conditions. Number of crosses examined: wild type = 10; *him-8(me4)* = 7; *sun-1p::TIR1; dpy-27::AID; him-8(me4)* −auxin = 7; *sun-1p::TIR1; dpy-27::AID; him-8(me4)* +auxin = 8.

**Fig. 4.**
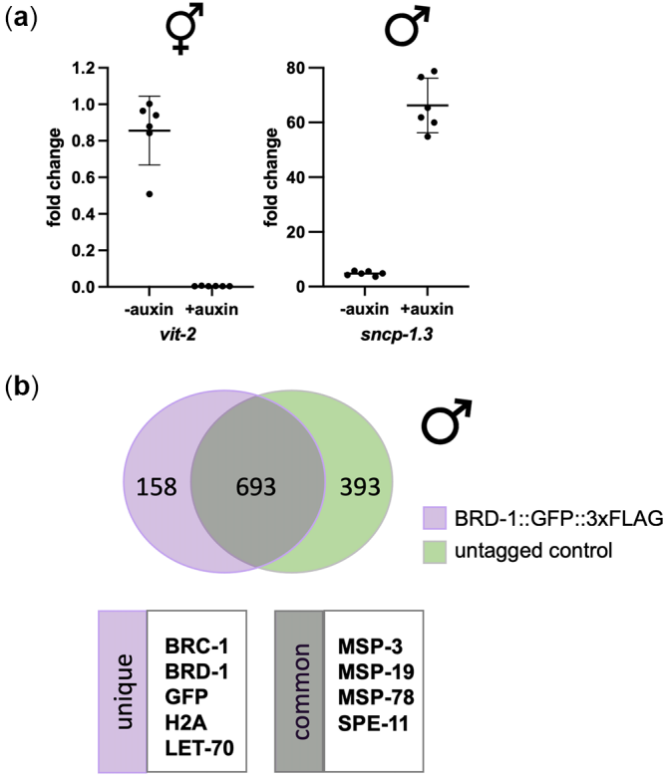
Molecular and biochemical analyses of males isolated following DPY-27 depletion. a) Fold change of mRNA expression for *vit-2* (hermaphrodite enriched) and *sncp-1.3* (male-enriched) *in sun-1p::TIR1; dpy-27::AID; him-8(me4)* in the absence and presence of auxin relative to wild type. Fold change was determined using the 2^−ΔΔCq^ method. *ama-1* (housekeeping) mRNA levels were used as a reference. Statistical comparisons by Mann–Whitney between − and + auxin for both *vit-2* and *sncp-1.3*, *P* = 0.0022. Raw Cq values and calculations are provided in Supplementary File 3. b) Mass spectrometry of peptides identified from male extracts (BRD-1 = 97.6% males; BRD-1::GFP::3xFLAG = 98.5% males). Venn diagram (generated by https://bioinfogp.cnb.csic.es/tools/venny/) shows unique peptides from BRD-1::GFP::3xFLAG and peptides from untagged control (BRD-1) with common peptides in middle. A small number of proteins with known interactions with BRC-1-BRD-1 (unique) as well as sperm proteins that are likely nonspecific interactions (common) are highlighted. The complete data set of nonspecific peptides and peptides unique to BRD-1::GFP::3xFLAG are available in Supplementary File 4.

Statistical analyses and figures were prepared using GraphPad Prism version 9.0 (GraphPad Software). Statistical comparisons of % males (Fig. 1c), RAD-51 (Fig. 2a) and GFP*::*COSA-1 foci numbers (Fig. 2b), meiotic divisions (Fig. 2c), sperm activation (Fig. 3b), progeny numbers (Fig. 3c), progeny viability (Fig. 3d), sperm competitiveness (Fig. 3e), and fold change in *vit-2* and *sncp-1.3* expression (Fig. 4a) were analyzed by Mann–Whitney. Detailed descriptions of statistical analyses are indicated in figure legends.

## Results and discussion

### Conditional degradation of DPY-27

The development of the auxin-inducible degradation system in *C. elegans* has facilitated analyses of many biological processes, including meiosis, spermatogenesis, mating, and aging (Zhang *et al.* 2015; Kasimatis *et al.* 2018; Ragle *et al.* 2020; Cahoon and Libuda 2021). The system requires the introduction of a short amino acid sequence, auxin-inducible degron (AID), onto a target protein, expression of the plant F-box protein TIR1, and the presence of the plant hormone auxin (Nishimura *et al.* 2009). TIR1 interacts with endogenous SKP1 and CUL1 proteins to form an SCF E3 ubiquitin ligase complex. In the presence of auxin, TIR1 recognizes and binds the AID sequence, leading to ubiquitination and subsequent degradation of the AID-tagged protein (Ruegger *et al.* 1998; Gray *et al.* 1999). We inserted an AID sequence together with an MYC tag at the C-terminus of DPY-27 (abbreviated as DPY-27*::*AID), which is a component of the DCC essential for embryonic development in hermaphrodites (Plenefisch *et al.* 1989), and generated *dpy-27::AID* strains that also expressed TIR1 under the control of the *sun-1* or *mex-5* promoter, which drive expression in germ cells and during early embryogenesis when dosage compensation is established (Zhang *et al.* 2015; Ashley *et al.* 2021). Additionally, these strains contain a mutation in *him-8*, which leads to the production of male self-progeny (Hodgkin *et al.* 1979; Fig. 1a). The AID tag does not interfere with DPY-27 function, as in the absence of auxin, there was no increase in male self-progeny (or decrease in hermaphrodite self-progeny) between AID-tagged and nontagged strains, indicating that DPY-27*::*AID is functional (*sun-1p::TIR1* or *mex-5p::TIR1; dpy-27::AID; him-8* vs *him-8*; Fig. 1c). In the presence of 1 mM auxin beginning at the L1 larval stage, the viable progeny of *sun-1p::TIR1; dpy-27::AID; him-8* hermaphrodites were almost exclusively males and the rare surviving hermaphrodites were dumpy, suggesting that dosage compensation was efficiently disrupted (Fig. 1, b and c). Treatment of L4 larvae also resulted in the production of almost all males (∼95%). In contrast, strains expressing the *mex-5* driven TIR1 did not result in as effective killing of hermaphrodite progenies (Fig. 1c). Therefore, all subsequent analyses were performed using strains expressing the *sun-1* promoter-driven TIR1.

### DPY-27 depletion does not affect male meiosis or spermiogenesis

To determine whether meiosis was affected in males isolated following DPY-27 degradation, we examined meiotic recombination and the meiotic divisions. We first monitored meiotic double-strand break (DSB) repair by examining the assembly and disassembly of the recombinase RAD-51 (Rinaldo *et al.* 2002) in the spatiotemporal organization of the *C. elegans* male germline using antibodies against RAD-51 (Colaiácovo *et al.* 2003; Checchi *et al.* 2014). RAD-51 loads onto resected DSBs destined for repair by homologous recombination beginning in leptotene/zygotene (transition zone) and is largely removed by mid-late pachytene. We had previously shown that *him-8* mutant males had wild-type levels of RAD-51 foci throughout the germline (Jaramillo-Lambert and Engebrecht 2010). Comparison of wild type, *him-8*, and *sun-1p::TIR1; dpy-27::AID; him-8* without and with auxin treatment revealed no differences, suggesting that DPY-27 does not play a role in the assembly or disassembly of RAD-51 on meiotic DSBs (Fig. 2a). To determine whether DSBs are accurately processed into crossovers, we monitored GFP*::*COSA-1, a cytological marker of crossover precursor sites (Yokoo *et al.* 2012). Wild-type males mostly exhibit 5 COSA-1 foci in pachytene nuclei, 1 on each of the 5 pairs of autosomes but not on the single *X* chromosome (Checchi *et al.* 2014). This pattern was unaltered by the degradation of DPY-27 (WT = 4.99 ± 0.30, *him-8*** **=** **4.97 ± 0.20, *sun-1p::TIR1; dpy-27::AID; him-8* -auxin = 5.00 ± 0.13, +auxin = 5.02 ± 0.17; Fig. 2b). Together, these results suggest that degradation of DPY-27 has no effect on DSB repair and crossover designation in male meiosis.

As germ cells progress down the gonad, they enter the division zone and undergo meiosis I and II divisions to produce haploid spermatids (Shakes *et al.* 2009). To examine the meiotic divisions, we labeled dissected and fixed gonads with antibodies directed against tubulin to visualize the spindle, in combination with DAPI to examine chromosome morphology. Both the spindles and chromosomes appeared similar in all genotypes and conditions examined (Fig. 2c). Additionally, we saw no difference in the number of meiotic divisions in the different strains and conditions *(*N2 = 13.5 ± 3.7; *him-8(me4)*=12.83 ± 2.1; *sun-1p::TIR1; dpy-27::AID; him-8(me4)—*auxin = 11.83 ± 2.3 and + auxin = 13.5 ± 1.9/gonad). Together, these results indicate that degradation of DPY-27 by the AID system does not affect male meiosis. A previous study showed that *dpy-27* depletion also had no effect on female meiosis (Tsai *et al.* 2008).

Following meiosis, sperm undergo postmeiotic differentiation, sperm activation, or spermiogenesis. During spermiogenesis, round spermatids become motile and competent for fertilization through fusion of membranous organelles with the plasma membrane and formation of a pseudopod (Ward and Carrel 1979). To examine sperm morphology and activation, we released spermatids from male worms and examined their morphology under differential interference contrast microscopy in WT, *him-8* and *sun-1p::TIR1; dpy-27::AID; him-8* in the absence and presence of auxin. We observed no difference in the morphology of the spermatids (Fig. 3a). We also examined sperm activation by releasing spermatids into a solution containing Pronase E, which has previously been shown to induce differentiation into spermatozoa (Singaravelu *et al.* 2011). Activation of spermatids achieved a level greater than 80% in all genotypes and conditions examined (Fig. 3, a and b).

To monitor the quantity, quality, and competitiveness of sperm produced following DPY-27 depletion, we performed mating experiments. To examine quantity and quality, we used the *fog-2(q71)* mutant to eliminate hermaphrodite spermatogenesis, rendering *XX* animals self-sterile (Schedl and Kimble 1988), so that the contribution of the male parent to progeny number and viability could be assessed unambiguously. We observed no difference between wild type, *him-8* and *sun-1p::TIR1; dpy-27::AID; him-8* in the absence or presence of auxin for either the total number of progeny sired or the ability to produce euploid gametes as indicated by embryonic viability (Fig. 3, c and d). The latter is important as *C. elegans* anucleate sperm are competent for fertilization (Sadler and Shakes 2000). We also performed a competition experiment by crossing males to *unc-119(ed3)* hermaphrodites for 16 h, transferred the hermaphrodites at 24 h intervals, and counted the total number of self and cross progeny at each time point. Male sperm from all genotypes and conditions were preferentially used over hermaphrodite self-sperm (Fig. 3d), suggesting that sperm derived following DPY-27 degradation maintains the capability to out compete hermaphrodite sperm (Ward and Carrel 1979; LaMunyon and Ward 1995). Together, these results indicate that spermiogenesis, sperm quality, quantity, and competitiveness are not affected following degradation of DPY-27 by the AID system.

### Molecular and biochemical studies using males collected following DPY-27 degradation

We used the *dyp-27::AID* system to monitor gene expression in the presence and absence of auxin. We first examined the expression of *vit-2*, 1 of 6 *vit* genes that encode vitellogenin (yolk proteins). *Vit* genes are expressed in the intestine of adult hermaphrodites, where the corresponding proteins are synthesized and then transported from the intestine into maturing oocytes (Kimble and Sharrock 1983; Grant and Hirsh 1999; Goszczynski *et al.* 2016). We collected adult worms from wild type (99.8% hermaphrodites/0.2% males) and *sun-1p::TIR1; dpy-27::AID; him-8* in the absence (60% hermaphrodites/40% males) and presence of auxin (0.5% hermaphrodites/99.5% males), extracted total RNA and analyzed *vit-2* expression using quantitative RT-PCR. We observed a small reduction in the expression of *vit-2* in *sun-1p::TIR1; dpy-27::AID; him-8* worms isolated from cultures grown in the absence of auxin compared to wild type (1.18-fold reduction), even though there was a 1.67-fold reduction in the number of hermaphrodites (Fig. 4a). This smaller than expected reduction in expression may be a consequence of the smaller body size of males compared to hermaphrodites, and/or due to inefficient collection of the male worms when a significant proportion of the population is hermaphrodites. On the other hand, we observed a 300-fold reduction of *vit-2* expression in *sun-1p::TIR1; dpy-27::AID; him-8* worms isolated from cultures grown in the presence of auxin compared to wild type (Fig. 4a). These results are consistent with strong enrichment of males in our cultures.

We next examined the expression of a male-specific transcription factor *snpc-1.3*. *snpc-1.3* drives male piRNA expression and is expressed in the male germline (Choi *et al.* 2021). We observed a 4-fold enrichment of *snpc-1.3* expression in *sun-1p::TIR1; dpy-27::AID; him-8* worms isolated from cultures grown in the absence of auxin and a 60-fold enrichment of *snpc-1.3* in the presence of auxin compared to wild type (Fig. 4a). Thus, our enrichment procedure facilitates analyses of sex-specific gene expression profiles.

Gene expression studies can be performed on a relatively small number of worms; however, biochemical analyses require more material. To determine the utility of our male enrichment system in biochemical analyses, we isolated large numbers of males following DPY-27 degradation from worms expressing wild-type BRD-1 or BRD-1*::*GFP*::*3xFLAG, a component of the *C. elegans* BRCA1-BARD1 (BRC-1-BRD-1) complex. BRC-1-BRD-1 is an E3 ubiquitin ligase enriched in nuclei throughout the germline and in embryos. The complex regulates several aspects of meiotic recombination in both oogenesis and spermatogenesis (Boulton *et al.* 2004; Polanowska *et al.* 2006; Janisiw *et al.* 2018; Li *et al.*^2018^, ^2020^).

We used magnetic anti-FLAG beads to pull-down proteins associated with BRD-1*::*GFP*::*3xFLAG from male whole worm lysates (see *Materials and Methods*). Mass spectrometry analyses of 2 independent pull-downs from both BRD-1 and BRD-1*::* GFP*::*3xFLAG lysates identified enrichment of sperm proteins (Fig. 4b). While these sperm proteins are likely not specific interactors of BRD-1, their identification is consistent with the strong enrichment of male worms following DPY-27 degradation. In the BRD-1*::*GFP*::*3xFLAG pull-downs, we specifically identified its binding partner BRC-1, histone H2A, a known target of mammalian BRCA1-BARD1 ubiquitinylation, **(**reviewed in Witus *et al.* 2021), and the E2 ubiquitin-conjugating enzyme LET-70/UBC-2, which had previously been shown to interact with the complex during DNA damage signaling in hermaphrodite worm extracts (Polanowska *et al.* 2006; Fig. 4b;Supplementary File 4). These results are consistent with a role for BRC-1-BRD-1 E3 ligase activity in male meiosis and demonstrate the utility of the system for examining protein interactions.

In conclusion, we describe a method to collect relatively pure populations of males that will facilitate future studies investigating how sex influences physiology. In combination with the large number of available mutants and continued improvement on the auxin-induced degradation system (Divekar *et al.* 2021; Hills-Muckey *et al.* 2022; Negishi *et al.* 2022), investigators can easily manipulate sex and determine the molecular processes that are differentially regulated in the sexes.

## Data availability

Strains and reagents are available upon request. JEL1197 was deposited at CGC. The authors affirm that all data necessary for confirming the conclusions of this article are represented fully within the article and its tables and figures.

Supplemental material is available at *G3* online.

## Supplementary Material

jkac085_Supplementary_File_1

jkac085_Supplementary_File_2

jkac085_Supplementary_File_3

jkac085_Supplementary_File_4
