# Supplementary material for: Inducible degradation of dosage compensation protein DPY-27 facilitates isolation of Caenorhabditis elegans males for molecular and biochemical analyses
Source: G3 (Bethesda). 2022 Apr 11;12(5):jkac085. doi: 10.1093/g3journal/jkac085 (PMC9073673; doi:10.1093/g3journal/jkac085)
Supplement: jkac085_Supplementary_File_1 [file jkac085_supplementary_file_1.docx]

| *dpy-27::AID::MYC* |
| --- |
| Guide: AATGTCTGCTTCTTCGCACA |
| Repair template: ATCGAAGAAGCAACTCCATCTCCACCACCAATTGTCGTTCAACGCAGAGTAAGACGATCCCGACACGGAGCATCGGGAGCCTCAATGCCTAAAGATCCAGCCAAACCTCCGGCCAAGGCACAAGTTGTGGGATGGCCACCGGTGAGATCATACCGGAAGAACGTGATGGTTTCCTGCCAAAAATCAAGCGGTGGCCCGGAGGCGGCGGCGTTCGTGAAGGGAGCCGGATCTGAACAAAAACTGATATCAGAAGAGGATCTGTAAATTGAAGTAATATATTTTAAACTAAATTTTGAAAAAAAAAAGAAAAC |
| Primers:  GTAAGAAGACGTCCGCGTCG  ACGTGATGCAAAGATATAGGGGT  525bps from knock-in animals vs 330bps from wild type. |

| *gfp(glo)::3xflag::cosa-1* |
| --- |
| Guide: AAGTGTCAATGTCAAGTTCT |
| Repair template:  GGTCAAAACTTTGTATTGGTCTCGCACCGAACCAGGTGTAGTACGGTAGATTTTTATTTATTTTATTTTATTTAACGACTTTTTATTCAATTTTCAACTCAAAAATCGACAAAATCAGTGAAAAATCGTGAAAACTGAACTGAAGTGTCAATGAGTAAAGGAGAAGAACTCTTTACCGGAGTCGTCCCAATTCTCGTCGAGCTCGACGGAGACGTAAACGGACACAAATTCTCGGTTTCCGGAGAAGGAGAAGGAGATGCTACTTATGGAAAACTCACCCTTAAATTCATTTGCACCACCGGAAAGCTCCCAGTGCCGTGGGTAAGTTTGTGATAATCCAATTTCAATTCGCAATGGTCATCGTTTTTTCAGCCGACCCTTGTGACTACATTCTGCTACGGAGTTCAATGCTTTTCCCGCTACCCAGACCACATGAAGCGTCACGATTTCTTCAAATCAGCCATGCCAGAAGGATACGTCCAGGAGCGAACAATTTTCTTCAAGGACGATGGAAACTACAAGACTCGTAAGTTTTTACTCCGCTTTTAACAATGGTTGTTTGACATCATTTTTTCAGGTGCTGAAGTCAAGTTTGAAGGAGATACTCTCGTGAACCGCATTGAGCTCAAGGGAATCGACTTCAAAGAAGATGGAAACATTCTTGGACACAAGCTCGAATACAACTATAACTCGCACAACGTGTATATCATGGCCGACAAGCAAAAAAATGGAATCAAGGTCGTAAGTTTGATGAAACGGTTTCGTCTTATATACACTAATGGTACTTTTCAGAACTTCAAGATTCGCCACAACATCGAAGACGGGTCGGTTCAACTCGCTGATCACTACCAGCAGAACACACCAATTGGAGATGGACCAGTCCTCCTCCCTGATAATCACTACCTTTCCACTCAATCCGCTCTTAGCAAGGATCCAAATGAGAAAAGAGATCACATGGTTCTTCTCGAGTTTGTCACCGCCGCCGGAATCACCCACGGAATGGACGAGTTGTACAAGGATTATAAAGACGATGACGATAAGCGTGACTACAAGGACGACGACGACAAGCGTGATTACAAGGATGACGATGACAAGAGAGGAGCATCGGGAGCCTCAGGAGCATCGATGAGTTCAAGCCGGTGAGTTGTCGTTTCAAAATAAAATGCGAACACTGCCTGTCACCATGTTTCTCGTGATAACAAATGCGTTTTTTACAGGTCACACCGCAAAAACACTTCAACTCTAGGTACACCTGCCGTATCAGCAGCGAATCAGGTTCGAAATATACC |
| Internal primer set:  GACAAGAGAGGAGCATCGGG  GAGCCGCCATACTCACGAAT  728bps from knock-in worms |
| External primer set:  CTTAGGCTCTGGTCTCGTGG  TTGTGCAATGCAATCCGTCC  1724 bps from knock-in worms and 749bps from wild type. |
