# Supplementary material for: Inducible degradation of dosage compensation protein DPY-27 facilitates isolation of Caenorhabditis elegans males for molecular and biochemical analyses
Source: G3 (Bethesda). 2022 Apr 11;12(5):jkac085. doi: 10.1093/g3journal/jkac085 (PMC9073673; doi:10.1093/g3journal/jkac085)
Supplement: jkac085_Supplementary_File_2 [file jkac085_supplementary_file_2.docx]

This protocol will generate 4-5ml of male worm pellet. Scale up or down as needed.

**Expansion of synchronized worms:**

- Transfer 6 L4 *sun-1p::TIR1; dpy-27::AID::MYC(xoe41); him-8(me4)* hermaphrodites onto each of 4 NGM plates seeded with *E. coli* OP50.
- When worms are freshly starved and mostly arrested as L1 larvae, chunk half of a plate onto a 150mm 8-times peptone-enriched (8P) plate seeded with *E. coli* NA22 (Nadarajan et al. 2016) for a total of 8 plates.
- Approximately 2½ days later at room temperature, most worms will have reached adulthood. Wash worms off the plates with water, and bleach using 0.5N NaOH and 1% bleach to kill worms and release embryos.
- Combine worms collected from four plates in one 50ml conical tube for a total of 2 tubes.
- wash with water once to remove most of the bacteria.
- Adjust total volume to 7ml with water.
- Add 2ml 5% bleach and 1ml 5N NaOH to the tube (final concentration: 1% bleach; 0.5N NaOH). Invert and vortex the tube constantly. Check beaching status frequently under the microscope. Solution will turn yellow over time and the whole process should take less than 8 minutes. Monitor carefully and do not overbleach, which will kill the embryos.
- Stop bleaching by immediately add M9 to 50ml.
- Centrifuge at 1300g for 1 minute to pellet embryos.
- Remove M9 by aspiration.
- Add M9 to 50ml. Invert the tube several times to wash the embryos.
- Centrifuge at 1300g for 1 minute to pellet embryos
- Wash embryos for a total of three times.
- Resuspend embryos in 3ml M9 and transfer to a new 50ml conical tube. Add M9 to 40ml and keep on a rocker for 24 hours to hatch.

**Auxin treatment:**

For each tube of synchronized worms, prepare 250ml liquid S medium in a 2L flask in the presence of 2.5mM K-NAA (auxin). Recipe for S medium is provided below as described in (Stiernagle 2006). High concentration of K-NAA is needed if worm density is high. Make sure enough concentrated *E. coli* is prepared as food supply (see below). Add 2-3L equivalent of overnight *E. coli* NA22 liquid culture to S medium.

- Wash synchronized worms once with S medium and transfer to flask.
- grow worms at 20°C in a shaker with vigorous shaking for three days.
- Add concentrated *E. coli* NA22 to flasks as needed on subsequent days.
- Collect worms from liquid culture and split worms into 5-6 50ml conical tubes. Bleach worms as above. Note that 2.5mM K-NAA is included in all liquid for this step.
- Keep embryos on a rocker for 24hr in the presence of 2.5mM K-NAA.

**Growth and collection of males:**

- Prepare up to 30 8P plates seed with *E.coli* NA22 at least 2 days before plating worms.
- Wash worms + dead embryos once with M9.
- Resuspend in 10ml M9.
- Seed 8P plates relatively evenly with worms + dead embryos. Do not overseed worms on plates.
- Males are ready to be collected for downstream analysis 2½ days later if kept at room temperature.
- Males are washed off plates with M9. Bacteria and dead embryos are removed by letting worms settle by gravity on ice and washing 3 times with M9.

S medium recipe (Stiernagle 2006)

- S Basal [5.85 g NaCl, 1 g K_2_ HPO_4_, 6 g KH_2_PO_4_. Add H_2_O to 1L. Sterilize by autoclaving.
- Add 1ml cholesterol (5 mg/ml in ethanol) to 1L S Basal before use.
- 1 M Potassium citrate pH 6.0 [20 g citric acid monohydrate, 293.5 g tri-potassium citrate monohydrate. Add H_2_O to 1L. Sterilize by autoclaving.
- Trace metals solution [1.86 g disodium EDTA, 0.69 g FeSO_4_ •7 H_2_O, 0.2 g MnCl2•4 H_2_O, 0.29 g ZnSO_4_ •7 H_2_O, 0.025 g CuSO_4_ •5 H_2_O, H_2_O to 1L. Sterilize by autoclaving. Store in the dark.
- To make 1L S medium, combine 1 liter S Basal with cholesterol, 10 ml 1 M potassium citrate pH 6, 10 ml trace metals solution, 3 ml sterile 1 M CaCl_2_, 3 ml sterile 1 M MgSO_4_.

Preparation of concentrated *E. coli* (NA22)

- Inoculate 1ml of saturated *E. coli* NA22 into 1L of Luria Broth and grow with shaking for 18hrs at 37^0^C. Prepare 4-6L for enough food supply.
- Pellet cells by centrifugation. Remove LB and flash freeze pellets in liquid nitrogen. Store at -80^0^C until ready to use.
- Thaw cells at room temperature and resuspend in 10-20mls of S medium supplied with 2.5mM K-NAA for transfer to liquid worm culture.

References

Nadarajan, S., F. Mohideen, Y. B. Tzur, N. Ferrandiz, O. Crawley, A. Montoya, P. Faull, A. P. Snijders, P. R. Cutillas, A. Jambhekar, M. D. Blower, E. Martinez-Perez, J. W. Harper, and M. P. Colaiacovo. 2016. The MAP kinase pathway coordinates crossover designation with disassembly of synaptonemal complex proteins during meiosis, *Elife*, 5: e12039.

Stiernagle, T. 2006. Maintenance of C. elegans. In: The C. elegans Research Community WormBook, editor. Wormbook. doi:10.1895/wormbook.1.101.1
